# The Effect of Plant Growth-Promoting Bacteria *Bacillus subtilis* IB-22 on the Hydraulic Conductivity and Abundance of PIP2 Aquaporins in the Roots of an Abscisic Acid-Deficient Barley Mutant

**DOI:** 10.3390/ijms251910706

**Published:** 2024-10-04

**Authors:** Zarina Akhtyamova, Tatiana Arkhipova, Guzel Sharipova, Ruslan Ivanov, Tatyana Nuzhnaya, Guzel Kudoyarova, Dmitry Veselov

**Affiliations:** Ufa Institute of Biology, Ufa Federal Research Centre, Russian Academy of Sciences, Prospekt Oktyabrya 69, 450054 Ufa, Russia; akhtyamovazarina@gmail.com (Z.A.); tnarkhipova@mail.ru (T.A.); g.v.sharipova@mail.ru (G.S.); ivanovirs@mail.ru (R.I.); tanyawww89@mail.ru (T.N.); guzel@anrb.ru (G.K.)

**Keywords:** *Hordeum vulgare*, abscisic acid, ABA-deficient mutant Az34, *Bacillus subtilis*, aquaporins

## Abstract

Little information is available on how rhizosphere bacteria affect abscisic acid (ABA) levels in plants and whether these bacterial effects are associated with improved plant water status. In this study, we tested the hypothesis that the stimulation of plant growth may be associated with the ability of ABA to increase the hydraulic conductivity of roots through the up-regulation of aquaporin. To do this, we studied the effect of bacteria capable of producing ABA on a barley mutant deficient in this hormone. Measurements of hydraulic conductivity of the ABA-deficient barley mutant Az34 showed that its tissues exhibited a reduced ability to conduct water, which correlated with lower ABA content in plants. The inoculation of *Bacillus subtilis* IB-22 stimulated the growth of both the mutant and its parent variety. Also, under the influence of bacteria, the ABA content in plants increased, and the increase was more significant in the mutant. This effect was accompanied by an increase in hydraulic conductivity in the roots of the ABA-deficient mutant, and immunolocalization using antibodies against PIP2;1 and PIP2;2 aquaporins revealed an increase in their abundance. Thus, the results obtained support the hypothesis about the importance of a sufficiently high ABA content in plants to maintain the abundance of aquaporins, hydraulic conductivity and the growth of barley plants.

## 1. Introduction

The ability of rhizosphere bacteria to either produce or catabolize phytohormones, as well as to influence their concentration in plants, is one of the main factors on which growth stimulation under the influence of such bacteria depends [1,2,3,4].

It is well known that most bacteria that promote plant growth produce auxins [5,6,7,8]. There is also a lot of information about the synthesis of cytokinins and gibberellins by these bacteria [9,10,11], but much less is known about their production of abscisic acid (ABA) [12,13,14], and information about the effect of rhizosphere bacteria on the level of ABA in plants is very limited and contradictory. Both an increase [15] and a decrease [16] in ABA levels were observed in inoculated plants. *Bacillus subtilis* IB-22 and *Pseudomonas mandelii* IB-Ki14 have been shown to induce ABA accumulation in wheat [17] and barley under salinity [13]. It has been suggested that the stimulation of plant growth in these cases is associated with the ability of ABA to increase the hydraulic conductivity of the roots due to the activation of aquaporins [18,19]. To test this assumption, it was important to study the effect of bacteria on ABA-deficient mutants. Hormonal mutants are widely used to elucidate the role of hormones in plant responses to growth-promoting bacteria. However, information on the effect of bacteria on ABA-deficient mutant plants is extremely limited. Only two studies have been conducted with ABA-deficient mutants of tomato [20] and barley [13]. These mutants in the cofactor of oxidase, which catalyzes the last stage of ABA synthesis from its aldehyde, are characterized by a reduced ability to accumulate this hormone [21]. It was previously shown [13] that the inoculation of the Az34 barley mutant with *Bacillus subtilis* IB-22 increased its water potential, the level of which was reduced in the non-inoculated mutant compared to the plants of the parent variety. It has been suggested that the restoration of normal water balance in mutant barley is likely due to an increase in hydraulic conductivity. The validity of this assumption is verified in this work, where we assessed the effect of *Bacillus subtilis* IB-22 on the abundance of aquaporins and the hydraulic conductivity of the roots of the Az34 barley mutant and plants of its parent variety Steptoe. The purpose of this work was to test a hypothesis that bacterial-induced increases in ABA levels in ABA-deficient plants lead to increased levels of aquaporins, thereby promoting increased hydraulic conductance and leaf water potential.

## 2. Results

**Figure 1:**
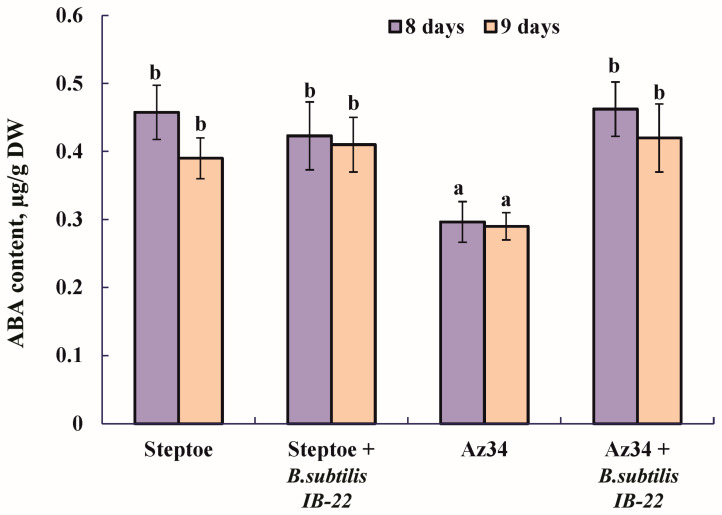
Effect of inoculation with *Bacillus subtilis* strain IB22 on the ABA content in 12- and 13-day-old whole plants of the ABA-deficient barley mutant Az34 and its parent variety Steptoe on the eighth and ninth days after bacterial treatment. Means ± SE are presented. Statistically different means (*n* = 10) are indicated by different letters (*p* < 0.05, ANOVA, Duncan’s test).

The measurement of ABA content in plants showed that, as expected, the level of this hormone was lower in the mutant compared to Steptoe plants (Figure 1). The introduction of bacteria into the rhizosphere of plants significantly increased ABA content in Az34 plants, and when the parental variety Steptoe was treated, a tendency for the content of this hormone to increase was observed (Figure 1).

**Figure 2:**
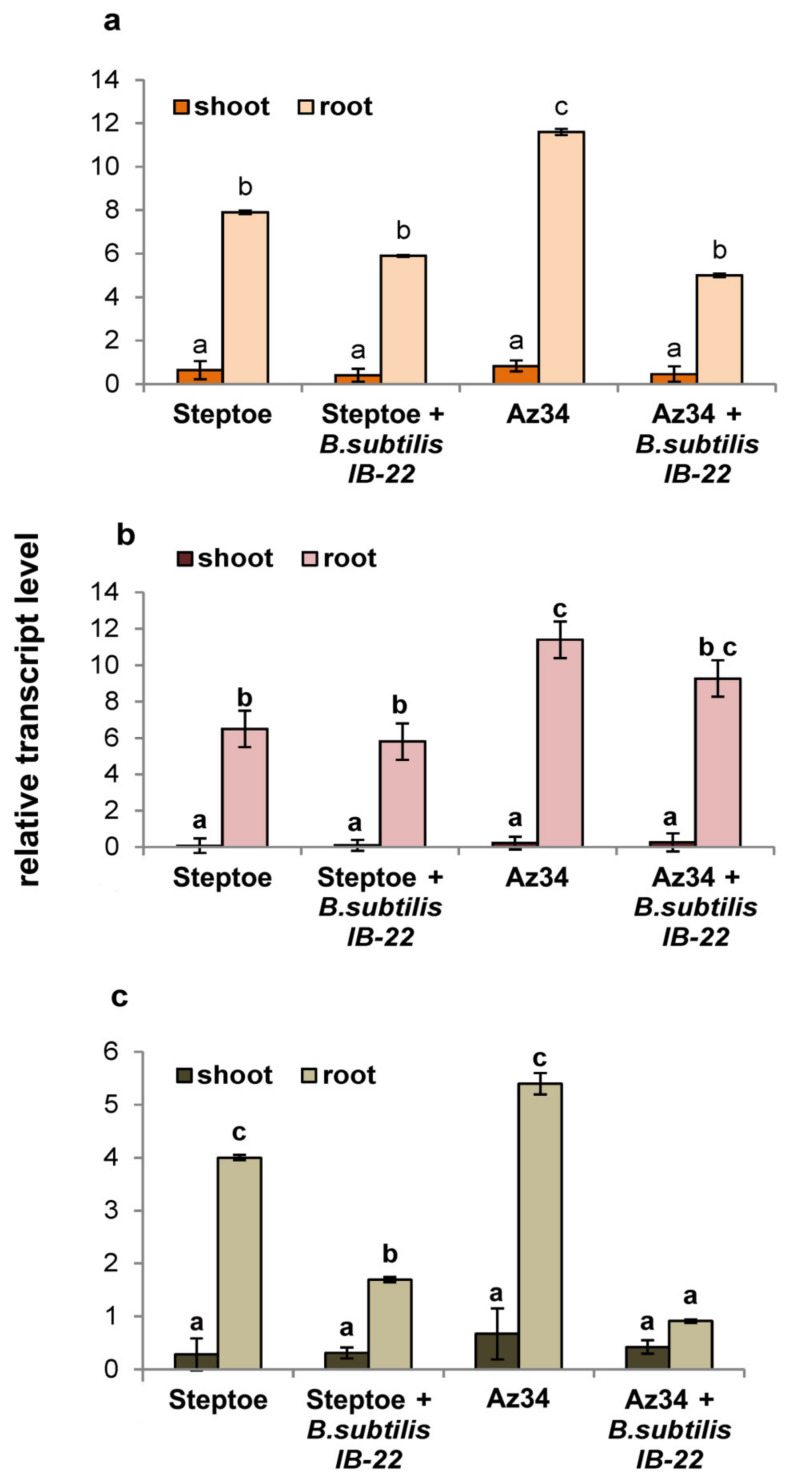
Effect of inoculation with *Bacillus subtilis* strain IB22 on the transcript level of the *HvNCED1* (**a**), *HvNCED2* (**b**), and *HvCYP707A1* (**c**) genes in shoots and roots of the 11-day-old ABA-deficient barley mutant Az34 and its parental cultivar Steptoe on the eighth day after bacterial treatment. The expression values of the genes responsible for ABA metabolism in barley are normalized relative to the barley gene *HvGADPH* encoding glyceraldehyde-3-phosphate dehydrogenase. Means ± SE are presented. Statistically different means (*n* = 6) are indicated by different letters (*p* < 0.05, ANOVA, Duncan’s test).

Figure 2 presents the results of assessing the level of transcripts of the *HvNCED1*, *HvNCED2* and *HvCYP707A1* genes, encoding enzymes that catalyze the rate-limiting step of ABA synthesis and the oxidative breakdown of ABA, respectively. As can be seen from Figure 2, the expression of genes responsible for ABA metabolism in roots is more than an order of magnitude higher than in plant shoots. Transcript levels of the *HvNCED1* and *HvNCED2* genes were higher in Az34 plants. The introduction of bacteria into the rhizo-sphere did not increase the expression level of these genes but led to a sharp decrease in the expression of *HvCYP707A1*. The degree of down-regulation of *HvCYP707A1* expression was higher in Az34 than in Steptoe (five-fold versus two-fold).

**Figure 3:**
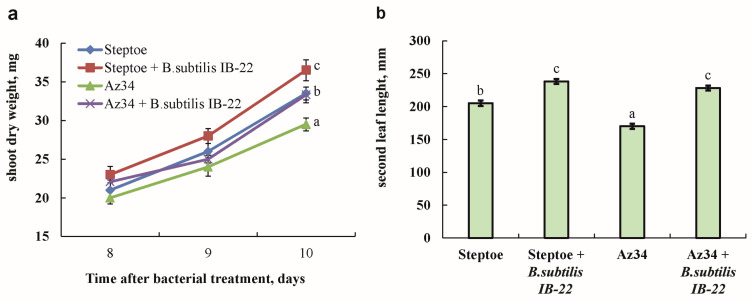
Dry mass of shoots measured on the eighth, ninth and tenth days after bacterial treatment (**a**) and the length of the second leaf of barley plants on the tenth day after bacterial treatment (**b**). Plants were 11 days old on the eighth day of bacterial treatment. Means ± SE are presented. Statistically different means (*n* = 25) are indicated by different letters (*p* < 0.05, ANOVA, Duncan’s test).

The fresh mass of shoots and the length of the second leaf of plants of the ABA-deficient mutant Az34 were smaller compared to plants of the parent variety Steptoe. Treatment with bacteria led to an increase in the mass of Steptoe and Az34 shoots by 19 and 24%, respectively (Figure 3).

The dry mass of the roots of mutant plants and their parental form did not differ significantly (Table 1).

Also, under the influence of bacteria, the length of the second leaf increased by 16% and 34% compared to untreated Steptoe and Az34 plants, respectively, and became the same in both genotypes. At the same time, the masses of the roots of both genotypes did not differ from each other, and bacterial treatment did not affect this indicator.

**Figure 4:**
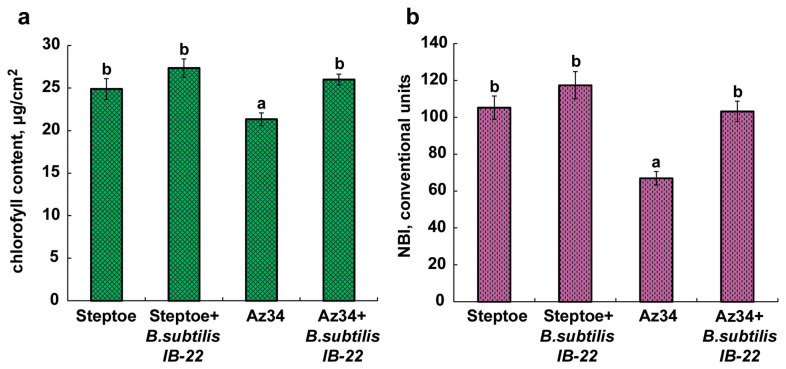
The content of the total chlorophylls (**a**) and the nitrogen balance index (NBI) (**b**) of 11 days old barley plants measured on the eighth day after bacterial treatment. Means ± SE are presented. Statistically different means (*n* = 30) are indicated by different letters (*p* < 0.05, ANOVA, Duncan’s test).

Plants of the ABA-deficient barley mutant Az34 were characterized by a lower chlorophyll content and nitrogen index compared to the parental variety. Under the influence of bacteria, these parameters in the parent variety did not change significantly, while in mutant plants, they increased to the level of the parent plants (Figure 4).

By the ninth day of the experiment, the hydraulic conductance of the ABA deficient mutant was about 1.5 times lower compared to plants of the parental genotype (Table 2). The response to bacterial treatment was genotype-specific: while the presence of *B. subtilis* IB-22 had a slight effect on hydraulic conductivity of the parental genotype, in the mutant, there was an approximate 1.5-fold increase.

**Figure 5:**
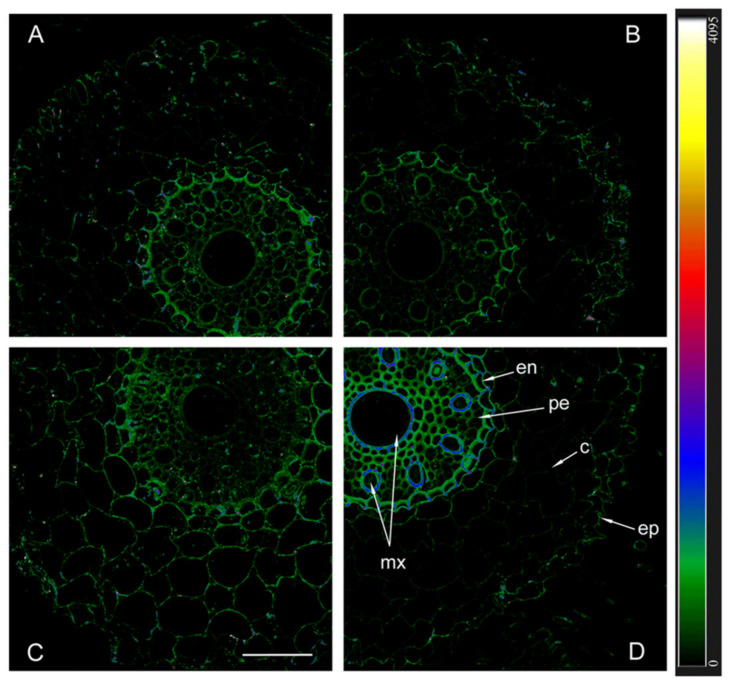
Immunohistochemical localization of HvPIP2;1 in the roots of 12-day-old barley plants of the Steptoe variety (**A**,**B**) and its ABA-deficient mutant Az34 (**C**,**D**) under control conditions (**A**,**C**) and under the influence of bacteria *B. subtilis* IB-22 (**B**,**D**) measured on the ninth day after bacterial treatment. Intensity of fluorescence is displayed as a color-coded heatmap. ep-epidermis, en-endodermis, mx-metaxylem, c-cortex, pe-pericycle. The line is 100 microns.

The immunolocalization of aquaporins with antibodies against HvPIP2;1 and their detection using secondary antibodies labeled with the fluorescent dye Alexa 555 revealed approximately the same level of these aquaporins in the plasma membrane of non-inoculated plants of both genotypes, as well as inoculated Steptoe plants (Figure 5).

Fluorescence was slightly more intense in the central cylinder compared to the cortex and most noticeable in the endoderm region of inoculated Az34. In contrast to other variants, fluorescence intensity was highest in inoculated mutant plants, indicating a high content of PIP2;1 aquaporins.

**Figure 6:**
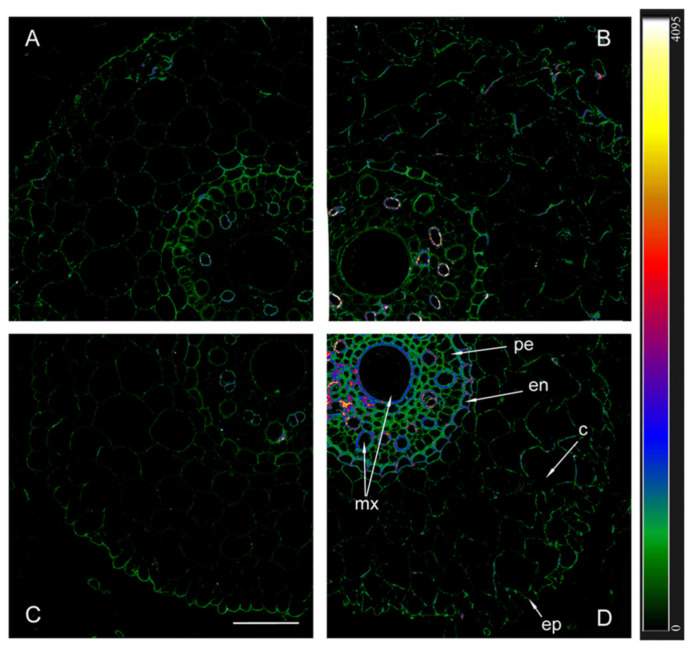
Immunohistochemical localization of HvPIP2;2 in the roots of 12-day-old barley plants of the Steptoe variety (**A**,**B**) and its ABA-deficient mutant Az34 (**C**,**D**) under control conditions (**A**,**C**) and under the influence of bacteria *B. subtilis* IB-22 (**B**,**D**) measured on the ninth after bacterial treatment. Intensity of fluorescence is displayed as a color-coded heatmap ep-epidermis, en-endodermis, mx-metaxylem, c-cortex, pe-pericycle. The line is 100 microns.

When using antibodies against HvPIP2;2, the fluorescence of the endoderm of uninoculated plants was noticeably lower in Az34 than in Steptoe (Figure 6). As in the case of HvPIP2;1, the highest level of HvPIP2;2 aquaporins was found in the plasmalemma of the cells of the central cylinder of inoculated mutant plants.

**Figure 7:**
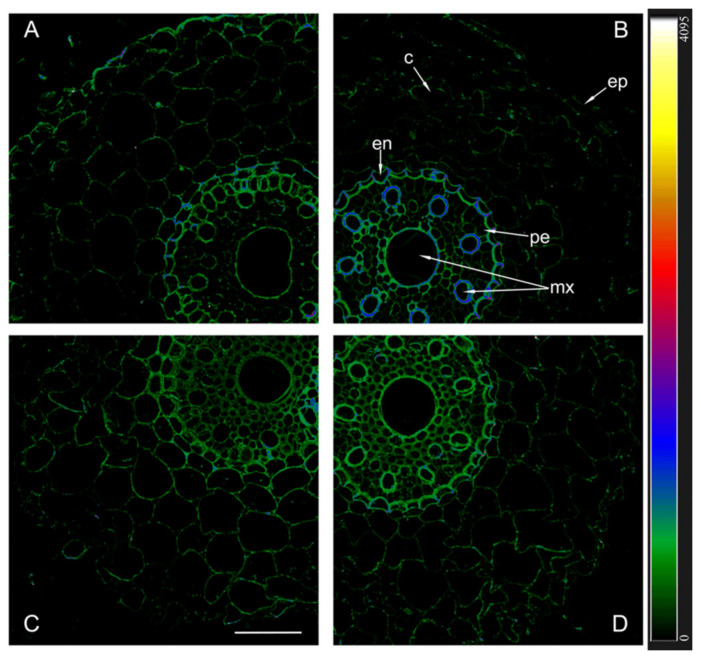
Immunohistochemical localization of HvPIP2;5 in the roots of 12-day-old barley plants of the Steptoe variety (**A**,**B**) and its ABA-deficient mutant Az34 (**C**,**D**) under control conditions (**A**,**C**) and under the influence of bacteria *B. subtilis* IB-22 (**B**,**D**) measured on the ninth after bacterial treatment. Intensity of fluorescence is displayed as a color-coded heatmap ep-epidermis, en-endoderm, mx-metaxylem, c-cortex, pe-pericycle. The line is 100 microns.

Immunostaining sections with antibodies against the aquaporins HvPIP2;5 revealed a different picture compared to what we found using antibodies against HvPIP2;1 and HvPIP2;2 (Figure 7). The brightest fluorescence in the region of the central cylinder was observed in sections of inoculated Steptoe roots.

## 3. Discussion

Plants of the ABA-deficient barley mutant not treated with bacteria differed from plants of the original genotype in having a smaller shoot mass and a shorter second leaf. These results may seem surprising since ABA has traditionally been thought to be a growth inhibitor [22,23,24]. However, arguments against ABA as a growth inhibitor were formulated in a paper by Humplík et al. [24]. This review suggested the requirement of this hormone for normal plant development and reported that ABA-deficient tomato mutants were smaller in size compared to wild-type plants [25,26]. The reduced ABA concentration in Az34 plants found in the present experiments is consistent with previously obtained data on abscisic acid deficiency in this mutant [19]. The low concentration of ABA in these barley plants is due to a mutation in the gene encoding the molybdenum cofactor of aldehyde oxidase, which catalyzes the synthesis of ABA from its aldehyde [27]. Despite the low activity of the corresponding enzyme, leading to a decrease in ABA concentration, this hormone was still present in mutant plants (Figure 1), which could be due to possible “gene leakage” [27] or the weakly expressed expression of some genes with a similar function [28]. The increased levels of *HvNCED1* and *HvNCED2* transcripts in the Az34 mutant compared with Steptoe found in the present experiments may also, to some extent, compensate for the low oxidation of ABA aldehyde (Figure 2). As a result, ABA concentration in the barley Az34 mutant was reduced to a lesser extent than in the ABA-deficient tomato mutant [13,20]. A deficiency of molybdenum cofactor in Az34 plants used in the present experiments affected not only oxidase, which catalyzes the last stage of ABA synthesis from its aldehyde, but also the NADH-dependent nitrate reductase enzyme—a key step in nitrogen assimilation. Consequently, the reduced growth rate detected in Az34 plants could be due to the impairment of nitrogen metabolism. However, the improvement of plant growth by Az34 bacteria confirms the importance of ABA changes in plants.

The inoculation of the Az34 rhizosphere in the present experiments increased ABA concentration. This effect may be due to the ability of the *B. subtilis* strain IB-22 to produce ABA [13]. It is also known from the literature that bacteria can change the level of ABA in plants, affecting the metabolism of this hormone in planta [20,29]. The introduction of bacteria into the rhizosphere did not increase the level of expression of the *HvNCED1* and *HvNCED2* genes but led to a sharp decline in the expression of *HvCYP707A1*, which could obviously contribute to the accumulation of ABA by reducing the activity of the oxidative catabolism of this hormone. Thus, the increase in ABA levels under the influence of bacteria could be associated both with the absorption of the hormone produced by bacteria and with a decrease in the breakdown of ABA under the influence of bacteria.

Measurements of hydraulic conductance in the ABA-deficient barley mutant showed that Az34 tissues have a reduced ability to conduct water. Since the influx of water into the shoot ensures plant growth, a decrease in the hydraulic conductivity of mutant tissues may be the reason for the lower rate of leaf elongation in the Az34 mutant compared to parental genotype Steptoe.

It is well known that ABA increases the hydraulic conductivity of tissues by influencing the level and activity of water channels—aquaporins [30,31]. Therefore, ABA deficiency in the mutant is the most likely cause of the decrease in the hydraulic conductivity of Az34 tissues. The results obtained in this work are consistent with previously obtained data, which also indicated the lower hydraulic conductivity of the roots of the Az34 plant compared to Steptoe [19]. This property of the roots of the ABA-deficient mutant has been previously explained by the weaker development of its root hair zone [19]. In the present study, the use of the immunohistochemical method revealed a reduced level of PIP2 aquaporins in the mutant, which has not been previously detected. The difference between the results of the present and earlier experiments is likely due to the fact that in the first case the plants were grown in sand, and in the second, they were grown in a hydroponic culture.

One way or another, ABA deficiency was accompanied by and, obviously, the cause of the decrease in the hydraulic conductivity and inhibition of shoot growth detected in Az34. The inoculation of *Bacillus subtilis* IB-22 stimulated the growth of both Az34 and Steptoe plants. The growth-stimulating effect of this strain was previously discovered in lettuce [32] and wheat plants [17]. These properties of bacteria were also recorded in the present work. In addition, the nitrogen balance index (NBI), which characterizes the ratio of chlorophyll (a + b) to flavonoids and is used to assess the nitrogen nutrition of crops [33], was higher in plants of both genotypes treated with bacteria (Figure 4b).

In the context of the problem we are discussing, it is important that the hydraulic conductivity of the roots of the ABA-deficient mutant increased under the influence of *Bacillus subtilis* IB-22 in accordance with the increase in ABA content in plants. An increase in the level of ABA under the influence of bacteria contributed to the normalization of leaf water potential in Az34 mutants under salt stress conditions [13]. This effect indicates an increase in water influx into the shoot under the influence of bacteria, which could be a consequence of an increase in hydraulic conductivity under the influence of increased ABA levels in inoculated mutant barley plants. However, the effect of bacteria on the hydraulic conductivity of the ABA-deficient mutant has not been previously assessed but was found in the present study.

The immunolocalization of aquaporins in plant roots showed that inoculation with *Bacillus subtilis* IB-22 led to an increase in the content of aquaporins PIP2;1 and PIP2;2 in plant Az34, which apparently contributed to an increase in hydraulic conductivity. In Steptoe plants, only the content of aquaporins PIP2;5 increased under the influence of bacteria, and, as a consequence, no increase in hydraulic conductivity was recorded in inoculated plants of this genotype. It was previously shown that the inoculation of a *Pseudomonas mandelii* strain into the rhizosphere of another barley variety (Prairie) increased not only the level of PIP2;5 but also other PIP2 aquaporins, which was accompanied by a significant increase in hydraulic conductivity [34]. Differences in plant responses were obviously associated with the characteristics of their genotype and bacterial species. Apparently, unlike Prairie and Az34, Steptoe plants were less sensitive to the effects of bacteria on ABA-dependent processes.

As noted in the Introduction, less attention was paid to the role of ABA in the interaction of bacteria and plants, compared with other hormones, and the available information is contradictory. Thus, there are publications stating that the stimulation of plant growth under the influence of bacteria is related to their ability to produce ABA [12], while in other works, growth stimulation was found when plants were inoculated with bacteria that can destroy this hormone, contributing to a decrease in its level in plants [35]. There is also contradictory information about the influence of bacteria on the content of ABA in plants, and the stimulation of plant growth under the influence of these bacteria was detected both with an increase and a decrease in the level of ABA in plants (see references above). In the first case, the beneficial effect of bacteria was explained by the fact that an accumulation of ABA contributes to the closure of stomata and water saving, and in the second—by the fact that a reduced level of ABA keeps stomata open, promoting gas exchange and photosynthesis. This duality may be due to the peculiarities of the growing conditions of plants, which determine what is more important for the plant in these conditions: to save water or to support photosynthesis. The novelty of the present research is in the accent of the importance of bacteria-induced changes in ABA for the regulation of hydraulic resistance. The effect of bacteria on the level and activity of aquaporins was previously studied [36], but it was not associated with a change in the level of ABA under the influence of bacteria.

## 4. Materials and Methods

The experiments were carried out on barley plants (*Hordeum vulgare* L.) cv. Steptoe and its ABA deficient mutant Az34.

### 4.1. Experimental Design

The sterilization of barley seeds and their stratification were performed as described previously [13]. Plants were grown at an irradiance of 400 µmol m^−2^ s^−1^ PAR, in a 14 h photoperiod and at 27/19 °C (day/night temperature) in 500 cm^3^ pots with sand, previously sterilized by calcination to prevent the introduction of unwanted bacteria. The sand was saturated with 50% Hoagland–Arnon solution to reach 90% of the total water capacity. To calculate the full water capacity of the substrate, dry sand used to fill the pots with holes was weighed and saturated with water. We waited until the pots stopped dripping at the holding (100%) capacity, measured the weight of pots with fully saturated sand and calculated the amount of water held by the sand. These values were used to calculate the amount of water to maintain the required level of sand humidity. A bacterial suspension (1 mL (10^8^ CFU mL^−1^) per seedling) was added into the rhizosphere simultaneously with planting by applying to the soil surface, and plants were watered daily with 10 mL of 10% Hoagland–Arnon added to each pot.

During the experiments, sand humidity was kept at 80% of the total water capacity. The amount of distilled water required for irrigation was calculated by weighing the containers with plants daily.

The mass of shoots and roots and the length of leaves were measured for 3 days, starting from the eighth day after bacterial inoculation.

### 4.2. Bacterial Strain and Culture Media

For the inoculation of the plant rhizosphere, we used the *Bacillus subtilis* IB-22 strain described in [32], cultivated as described [13].

### 4.3. ABA Assay

Sampling the plants for the ABA assay was carried out on the eighth and ninth day after bacterial inoculation, when plants were 11 and 12 days old, correspondingly. The extraction of the hormone using a modified procedure and its immunoassay with specific antibodies against ABA were carried out as described previously [13]. The reliability of the method was confirmed by a comparison between the results of immune- and physicochemical analyses [37].

### 4.4. The Content of the Sum of Chlorophylls and Nitrogen Index in the Epidermis of the First Barley Leaf

The content of the sum of chlorophylls and nitrogen index in the epidermis of the first barley leaf was determined 8 days after bacterial treatment using the DUALEX SCIENTIFIC+ device (FORCE-A, Orsay, France).

### 4.5. Parameters of Water Relations

Transpiration and water potential were measured as described [19] on the ninth day after bacterial inoculation. Hydraulic conductance, which is a measure of the efficiency of bulk flow through a material and defined as the flow rate (transpiration) per unit pressure driving force (water potential gradient between leaf and soil), was calculated, as described [34].

### 4.6. RNA Extraction and Quantitative Real-Time Polymerase Chain Reaction (qPCR)

Sampling plants for RNA extraction was carried out on the eighth day after bacterial treatment. The procedures for RNA isolation, selection of primers, and qPCR were described by us previously [13].

### 4.7. Immunolocalization of Aquaporins

Sampling root sections for the immunolocalization of PIP2 aquaporins with specific antibodies was performed on the ninth day after bacterial treatment. The fixation of tissues, dehydration, embedding, preparation of sections, treatment with rabbit antibodies against PIP2 and second antibodies against rabbit immunoglobulins labeled with fluorescent dye, as well as the registration of fluorescence with confocal microscopy, have been described previously [34].

### 4.8. Statistics

The data were processed using the Statistica version 10 software (Statsoft, Moscow, Russia) and MS Excel version 14.0 (Microsoft, Redmond, WA, USA) programs. In the figures and tables, data are presented as mean ± standard error. The significance of differences was assessed by ANOVA followed by Duncan’s test (*p* ≤ 0.05).

## 5. Conclusions

The results obtained in this work indicate the requirement of ABA to maintain hydraulic conductivity and plant growth. A deficiency of this hormone in mutant barley led to a decrease in hydraulic conductivity compared to plants of the parental genotype, which was accompanied by a slower accumulation of plant biomass. The compensation of ABA deficiency in the Az34 mutant under the influence of the inoculation of bacteria capable of producing ABA, as well as an increase in its content in the plant due to the influence on the metabolism of this hormone in plants, contributed to an increase in the hydraulic conductivity of plants due to an increase in the abundance of PIP2 aquaporins and the manifestation of the growth-stimulating effect of bacteria. Bacteria capable of synthesizing ABA may have a beneficial effect on water relations in varieties with genetically controlled low levels of production of this hormone. Preparations of such bacteria can be recommended for use in agriculture.

## Figures and Tables

**Table 1:** Dry mass of roots. Means ± SE are presented. Statistically different means (*n* = 25) are indicated by different letters (*p* < 0.05, ANOVA, Duncan’s test). Age of plants is indicated in the brackets.

Genotypes, Treatment	Time after Bacterial Treatment, Days		
	8 (11 Days Old)	9 (12 Days Old)	10 (13 Days Old)
Steptoe	21.4 ± 0.6 ^a^	24.1 ± 0.9 ^b^	27.5 ± 0.8 ^c^
Steptoe, *B. subtilis* IB-22	18.9 ± 0.3 ^a^	22.4 ± 0.5 ^b^	24.6 ± 0.7 ^c^
Az34	20.4 ± 0.5 ^a^	22.7 ± 0.7 ^b^	26.5 ± 1.0 ^c^
Az34, *B. subtilis* IB-22	18.2 ± 0.4 ^a^	21.3 ± 0.4 ^b^	24.0 ± 0.9 ^c^

**Table 2:** Transpiration, soil and leaf water potential and hydraulic conductance of 12-day-old barley plants measured on ninth day after plant treatment with *B. subtilis* IB-22. Means ± SE are presented. Statistically different means (*n* = 10) are indicated by different letters (*p* < 0.05, ANOVA, Duncan’s test).

Genotypes, Treatment	Transpiration, mg of Water Per Plant h^−1^	WP Soil, MPa	WP Leaf, MPa	Hydraulic Conductance, mg of Water Per Plant h^−1^ MPa^−1^
Steptoe	152 ± 7 ^a^	−0.01 ± 0.01 ^a^	−0.36 ± 0.05 ^a^	428 ± 39 ^b^
Steptoe, *B. subtilis* IB-22	156 ± 5 ^a^	−0.09 ± 0.03 ^a^	−0.45 ± 0.07 ^ab^	437 ± 41 ^b^
Az34	160 ± 8 ^a^	−0.02 ± 0.01 ^a^	−0.6 ± 0.08 ^b^	275 ± 25 ^a^
Az34, *B. subtilis* IB-22	161 ± 7 ^a^	−0.05 ± 0.03 ^a^	−0.43 ± 0.06 ^ab^	429 ± 41 ^b^

## Data Availability

The data presented in this study are available in the graphs and tables provided in the manuscript.
